# Nursing and equity: challenges of Primary Health Care in approaching transgender people

**DOI:** 10.1590/0034-7167.202578suppl0401

**Published:** 2025-09-01

**Authors:** Sergio Neder Rocha, Fábio de Souza Terra, Patrícia Scotini Freitas

**Affiliations:** I Universidade Federal de Alfenas. Alfenas, Minas Gerais, Brazil

Health is a fundamental right of every citizen and a duty of the State, guaranteed by the Federal Constitution and operationalized by the Brazilian Healthcare System (in Portuguese, *Sistema Único de Saúde* - SUS), whose principles of universality, comprehensiveness and equity guide care actions^(1)^. However, when it comes to the transgender population, access to healthcare services is still far from being equitable. It is worth noting that structural and institutional transphobia, combined with the invisibility of this population, still represents a major obstacle to healthcare in healthcare services, especially Primary Health Care (PHC)^(2)^.

In this context, PHC becomes a strategic setting for addressing inequities, since it is users’ gateway to the SUS, and its mission is to promote continuous, humanized, accessible care that is close to people’s reality. It is at this level of health care that nurses act as agents of transformation, being responsible for welcoming, listening, guiding, educating and coordinating care in a network. The role of nurses, therefore, goes far beyond clinical practice: they take on an ethical and political role in the construction of inclusive and anti-discriminatory practices^(3)^.

Despite legal and regulatory advances, such as the creation of the Brazilian National Policy for Comprehensive Health for the LGBT Population^(1)^, there is still a gap between theory and practice. Many healthcare professionals, due to a lack of adequate training or prejudiced attitudes, make it difficult or impossible for the transgender population to access a wide range of services. Negligence can take subtle forms, such as the improper use of a person’s legal name, refusal to provide specialized care, or disregard for this population’s specific needs.

Nurses, as members of the primary care team, are often the first healthcare professionals that transgender people come into contact with when they are approached. Their attentive and sensitive approach can be decisive in building a therapeutic bond. It is up to these professionals to ensure the use of their social name, to welcome them with empathy, to respect their ways of life and to recognize the multiple vulnerabilities faced by these people, which range from family exclusion to exposure to violence and unemployment^(4)^.

In addition to providing a humanized welcome, nurses must be able to provide qualified technical assistance that takes into account the specificities of transgender people’s health. This includes, for instance, monitoring the use of hormone therapy, paying attention to adverse effects and drug interactions, performing preventive exams appropriate to each person’s anatomy, and providing guidance on safe sexual practices, self-care, and mental health. It is also necessary to understand that not all transgender people want or have access to body transition procedures, which requires sensitivity and a lack of judgment^(2)^.

Therefore, nurses’ work must be coordinated with health promotion, disease prevention, and also strengthening the role of the transgender population in the construction of their therapeutic projects. Care must be shared, respecting a person’s autonomy and their active participation in decisions about their body and health. To this end, it is essential that professionals, especially nurses, continually train themselves, breaking with stigmatized views and updating their knowledge based on scientific evidence and successful experiences^(4)^.

Another crucial aspect that deserves to be addressed refers to coping with transphobia in healthcare services. Nurses, as team leaders and reference points for the community, have the duty to promote the continuing education of other professionals, encourage debates on gender diversity, sexual orientation and human rights, and act as advocates for anti-discrimination practices. This includes mediating conflicts, developing reception protocols and strengthening partnerships with social movements and institutions that work with the transgender population^(5)^.

In this sense, the role of nurses in PHC should be considered as an exercise in citizenship and an ethical commitment to life. By recognizing that health is also about belonging, visibility and respect, nurses contribute to the construction of a fairer society, in which all people have the right to receive equal and comprehensive care. Caring for the transgender population is a professional duty and a reaffirmation of the principles that underpin the SUS^(6)^.

Thus, for public policy to become everyday practice, healthcare must go beyond the walls of the unit and become part of the territory as a tool for social transformation. Nurses are an essential link in this process, as their listening, welcoming and clinical and political actions are capable of producing significant changes in the lives of people who, for so long, have been silenced and made invisible.

Finally, it is important to emphasize that building a more inclusive healthcare system requires courage to face prejudices, learn from diversity and a commitment to transform reality. Each nurse should recognize themselves as a fundamental part of this change.
