# Supplementary material for: Nursing and equity: challenges of Primary Health Care in approaching transgender people
Source: Rev Bras Enferm. 2025 Sep 1;78(Suppl 4):e78suppl0401. doi: 10.1590/0034-7167.202578suppl0401 (PMC12419774; doi:10.1590/0034-7167.202578suppl0401)
Supplement: Translation

# Translation: Enfermagem e equidade: desafios da Atenção Primária à Saúde na abordagem de pessoas transgênero

**DOI:** 10.1590/0034-7167.202578suppl0401pt

**Authors:** Sergio Neder Rocha, Fábio de Souza Terra, Patrícia Scotini Freitas

**Affiliations:** I Universidade Federal de Alfenas. Alfenas, Minas Gerais, Brazil

A saúde é direito fundamental de todo cidadão e dever do Estado, garantido pela Constituição Federal e operacionalizado pelo Sistema Único de Saúde (SUS), cujos princípios da universalidade, integralidade e equidade orientam as ações de cuidado^(1)^. Contudo, quando se trata da população transgênero, o acesso aos serviços de saúde ainda está longe de ser equitativo. Destaca-se que a transfobia estrutural e institucional, somada à invisibilidade dessa população, ainda representa um grande entrave ao cuidado em saúde nos serviços de saúde, em especial a Atenção Primária à Saúde (APS)^(2)^.

Neste contexto, a APS se torna um cenário estratégico para o enfrentamento das iniquidades, uma vez que é a porta de entrada do usuário ao SUS, e tem como missão promover o cuidado contínuo, humanizado, acessível e próximo à realidade das pessoas. É nesse nível de atenção à saúde que o(a) enfermeiro(a) atua como agente transformador, sendo responsável por acolher, escutar, orientar, educar e articular o cuidado em rede. O papel do(a) enfermeiro(a), portanto, vai muito além da prática clínica: ele(ela) assume uma função ética e política na construção de práticas inclusivas e antidiscriminatórias^(3)^.

Apesar dos avanços legais e normativos, como a criação da Política Nacional de Saúde Integral da População LGBT^(1)^, ainda se observa um descompasso entre a teoria e a prática assistencial. Muitos profissionais de saúde, por falta de formação adequada ou por atitudes preconceituosas, dificultam ou inviabilizam o acesso da população transgênero aos mais diversos tipos de serviços. A negligência pode assumir formas sutis, como o uso indevido do nome civil, a recusa em prestar atendimento especializado ou a desconsideração das necessidades específicas dessa população.

O(a) enfermeiro(a), como integrante da equipe de saúde da APS, é, muitas vezes, o(a) primeiro(a) profissional de saúde com quem a pessoa transgênero tem contato ao ser abordada. Seu olhar atento e sensível pode ser decisivo para a construção de um vínculo terapêutico. Cabe a esse profissional assegurar o uso do nome social, acolher com empatia, respeitar os modos de vida e reconhecer as múltiplas vulnerabilidades enfrentadas por essas pessoas, que vão desde a exclusão familiar até a exposição à violência e ao desemprego^(4)^.

Além do acolhimento humanizado, o(a) enfermeiro(a) deve estar apto a prestar uma assistência técnica qualificada que contemple as especificidades da saúde de pessoas transgênero. Isso inclui, por exemplo, o acompanhamento no uso de hormonioterapia, com atenção aos efeitos adversos e à interação medicamentosa, a realização de exames preventivos adequados à anatomia de cada pessoa, e a orientação sobre práticas seguras de sexo, autocuidado e saúde mental. Também é necessário compreender que nem todas as pessoas transgênero desejam ou têm acesso a procedimentos de transição corporal, o que exige sensibilidade e ausência de julgamentos^(2)^.

Com isso, a atuação do(a) enfermeiro(a) deve estar articulada com a promoção da saúde, a prevenção de agravos e também o fortalecimento do protagonismo da população transgênero na construção de seus projetos terapêuticos. O cuidado deve ser compartilhado, respeitando a autonomia da pessoa e sua participação ativa nas decisões sobre seu corpo e sua saúde. Para isso, é fundamental que os profissionais, principalmente os de enfermagem, capacitem-se continuamente, rompendo com visões estigmatizadas e atualizando seus saberes com base em evidências científicas e experiências exitosas^(4)^.

Outro aspecto crucial e que merece ser abordado se refere ao enfrentamento da transfobia nos serviços de saúde. O(a) enfermeiro(a), enquanto líder de equipe e referência para a comunidade, tem o dever de promover a educação permanente dos demais profissionais, fomentar debates sobre diversidade de gênero, orientação sexual e direitos humanos, e atuar como defensor de práticas antidiscriminatórias. Isso inclui a mediação de conflitos, a construção de protocolos de acolhimento e o fortalecimento de parcerias com movimentos sociais e instituições que atuam com a população transgênero^(5)^.

Nesse sentido, a atuação do(a) enfermeiro(a) na APS deve ser pensada como um exercício de cidadania e um compromisso ético com a vida. Ao reconhecer que saúde também é pertencimento, visibilidade e respeito, o(a) enfermeiro(a) contribui para a construção de uma sociedade mais justa, em que todas as pessoas tenham direito de serem atendidas com a mesma oportunidade e de forma integral. Cuidar da população transgênero é dever profissional e uma reafirmação dos princípios que fundam o SUS^(6)^.

Assim, para que a política pública se transforme em prática cotidiana, é preciso que o cuidado em saúde ultrapasse os muros da unidade e se insira no território como ferramenta de transformação social. O(a) enfermeiro(a) é um elo essencial nesse processo, pois sua escuta, seu acolhimento e sua atuação clínica e política são capazes de produzir mudanças significativas na vida de pessoas que, por tanto tempo, ainda são silenciadas e invisibilizadas.

Por fim, faz-se necessário enfatizar que a construção de um sistema de saúde mais inclusivo exige coragem para enfrentar preconceitos, aprender com a diversidade e o compromisso para transformar a realidade. Que cada enfermeiro(a) se reconheça como parte fundamental dessa mudança.

# Translation: Enfermería y equidad: desafíos de la Atención Primaria de Salud en el abordaje de las personas transgénero

**DOI:** 10.1590/0034-7167.202578suppl0401es

**Authors:** Sergio Neder Rocha, Fábio de Souza Terra, Patrícia Scotini Freitas

**Affiliations:** I Universidade Federal de Alfenas. Alfenas, Minas Gerais, Brazil

La salud es un derecho fundamental de todo ciudadano y un deber del Estado, garantizado por la Constitución Federal y operativizado por el Sistema Único de Salud (en portugués, *Sistema Único de Saúde* - SUS), cuyos principios de universalidad, integralidad y equidad orientan las acciones de atención^(1)^. Sin embargo, en el caso de la población transgénero, el acceso a los servicios de salud aún dista mucho de ser equitativo. Cabe destacar que la transfobia estructural e institucional, sumada a la invisibilidad de esta población, sigue representando un importante obstáculo para la atención en los servicios de salud, especialmente en la Atención Primaria de Salud (APS)^(2)^.

En este contexto, la APS se convierte en un espacio estratégico para abordar las inequidades, ya que es la puerta de entrada del usuario al SUS, y su misión es promover una atención continua, humanizada, accesible y cercana a la realidad de las personas. Es en este nivel de atención médica donde los(las) enfermeros(as) actúan como agentes de transformación, siendo responsables de acoger, escuchar, guiar, educar y coordinar la atención en red. El rol de los(las) enfermeros(as), por lo tanto, trasciende la práctica clínica: asumen un rol ético y político en la construcción de prácticas inclusivas y antidiscriminatorias^(3)^.

A pesar de los avances legales y regulatorios, como la creación de la Política Nacional de Salud Integral para la Población LGBT^(1)^, aún existe una brecha entre la teoría y la práctica. Muchos profesionales de la salud, debido a la falta de capacitación adecuada o a actitudes prejuiciosas, dificultan o imposibilitan el acceso de la población transgénero a una amplia gama de servicios. La negligencia puede manifestarse de forma sutil, como el uso indebido del nombre legal de una persona, la negativa a brindar atención especializada o la indiferencia hacia las necesidades específicas de esta población.

El personal de enfermería, como miembros del equipo de atención primaria, suele ser el primer profesional de la salud con el que las personas transgénero entran en contacto cuando se les acerca. Su enfoque atento y sensible puede ser decisivo para construir un vínculo terapéutico. Es responsabilidad de estos profesionales garantizar el uso de su nombre social, acogerlos con empatía, respetar sus formas de vida y reconocer las múltiples vulnerabilidades que enfrentan estas personas, que van desde la exclusión familiar hasta la exposición a la violencia y el desempleo^(4)^.

Además de brindar una acogida humanizada, el personal de enfermería debe ser capaz de brindar asistencia técnica cualificada que tenga en cuenta las particularidades de la salud de las personas transgénero. Esto incluye, por ejemplo, supervisar el uso de la terapia hormonal, prestar atención a los efectos adversos y las interacciones farmacológicas, realizar exámenes preventivos adaptados a la anatomía de cada persona y brindar orientación sobre prácticas sexuales seguras, autocuidado y salud mental. También es necesario comprender que no todas las personas transgénero desean o tienen acceso a procedimientos de transición corporal, lo que requiere sensibilidad y no tener prejuicios^(2)^.

Por lo tanto, la labor de enfermería debe coordinarse con la promoción de la salud, la prevención de enfermedades y el fortalecimiento del rol de la población transgénero en la construcción de sus proyectos terapéuticos. El cuidado debe ser compartido, respetando la autonomía de la persona y su participación activa en las decisiones sobre su cuerpo y salud. Para ello, es fundamental que los profesionales, especialmente las enfermeras, se capaciten continuamente, rompiendo con las visiones estigmatizadas y actualizando sus conocimientos con base en la evidencia científica y experiencias exitosas^(4)^.

Otro aspecto crucial que merece ser abordado se refiere a la lucha contra la transfobia en los servicios de salud. Los(las) enfermeros(as), como líderes de equipo y referentes para la comunidad, tienen el deber de promover la formación continua de otros profesionales, fomentar debates sobre diversidad de género, orientación sexual y derechos humanos, y actuar como defensoras de prácticas antidiscriminatorias. Esto incluye la mediación en conflictos, el desarrollo de protocolos de acogida y el fortalecimiento de las alianzas con movimientos sociales e instituciones que trabajan con la población transgénero^(5)^.

En este sentido, el rol de los(las) enfermeros(as) en la APS debe considerarse un ejercicio de ciudadanía y un compromiso ético con la vida. Al reconocer que la salud también implica pertenencia, visibilidad y respeto, los(las) enfermeros(as) contribuyen a la construcción de una sociedad más justa, donde todas las personas tienen derecho a recibir una atención igualitaria e integral. Atender a la población transgénero es un deber profesional y una reafirmación de los principios que sustentan el SUS^(6)^.

Así, para que las políticas públicas se conviertan en una práctica cotidiana, la atención sanitaria debe trascender los muros de la unidad y formar parte del territorio como herramienta de transformación social. Los(las) enfermeros(as) son un eslabón esencial en este proceso, ya que su escucha, acogida y sus acciones clínicas y políticas son capaces de generar cambios significativos en la vida de personas que, durante tanto tiempo, han sido silenciadas e invisibilizadas.

Finalmente, es importante destacar que construir un sistema de salud más inclusivo requiere valentía para afrontar los prejuicios, aprender de la diversidad y el compromiso de transformar la realidad. Cada enfermero(a) debe reconocerse como parte fundamental de este cambio.
